# The effects of cocoa flavanols on indices of muscle recovery and exercise performance: a narrative review

**DOI:** 10.1186/s13102-021-00319-8

**Published:** 2021-08-14

**Authors:** Liam D. Corr, Adam Field, Deborah Pufal, Tom Clifford, Liam D. Harper, Robert J. Naughton

**Affiliations:** 1 School of Human and Health Sciences, University of Huddersfield, Huddersfield, UK; 2 School of Sport, Health, and Exercise Sciences, Loughborough University, Loughborough, UK

**Keywords:** Dark chocolate, Muscle damage, Fatigue, Muscle function, Oxidative stress, Inflammation

## Abstract

**Abstract:**

Exercise-induced muscle damage (EIMD) is associated with oxidative stress and inflammation, muscle soreness, and reductions in muscle function. Cocoa flavanols (CF) are (poly)phenols with antioxidant and anti-inflammatory effects and thus may attenuate symptoms of EIMD. The purpose of this narrative review was to collate and evaluate the current literature investigating the effect of CF supplementation on markers of exercise-induced oxidative stress and inflammation, as well as changes in muscle function, perceived soreness, and exercise performance. Acute and sub-chronic intake of CF reduces oxidative stress resulting from exercise. Evidence for the effect of CF on exercise-induced inflammation is lacking and the impact on muscle function, perceived soreness and exercise performance is inconsistent across studies. Supplementation of CF may reduce exercise-induced oxidative stress, with potential for delaying fatigue, but more evidence is required for any definitive conclusions on the impact of CF on markers of EIMD.

**Graphic abstract:**

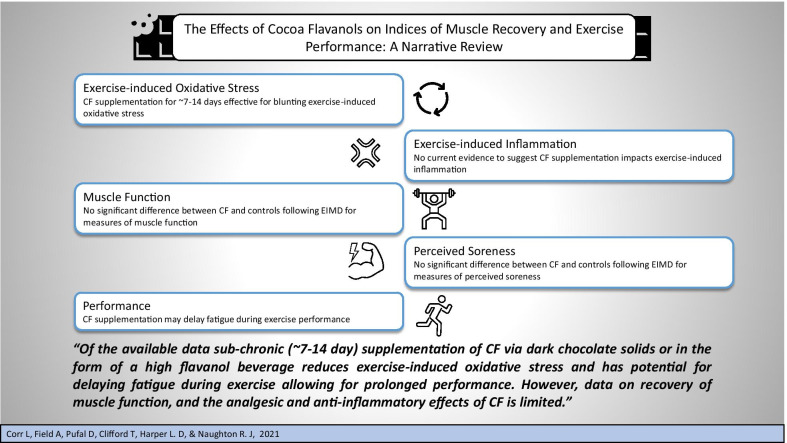

## Background

Exercise-induced muscle damage (EIMD) is associated with various negative symptoms, such as delayed onset muscle soreness, impaired muscle function, and increased inflammation [1, 2]. Consequently, the use of recovery interventions purported to accelerate recovery has become increasingly prevalent. There is an emerging interest in the effects of the non-nutritive compounds (poly)phenols as recovery aids following strenuous exercise. As such their popularity as a nutritional aid has increased in athletes and recreational exercisers, likely because these plant-based bioactive compounds have numerous additional health benefits [3].

The term (poly)phenol refers to a variety of bioactive compounds including flavonoids, stilbenes, phenolic acids and lignans [4]. The largest subclass, flavonoids, can be further classified into flavonols, flavanols, flavanones, anthocyanins, flavones and isoflavones. Of these subclasses, the majority of research has focused on flavanols with particular attention on cocoa, not only because of the palatability of chocolate [5] but due to the high proportion of monomers such as catechin, epicatechin and gallocatechin; collectively referred to as cocoa flavanols (CF). These monomers are found in the largest quantities in cocoa when compared with other flavanol containing foodstuffs such as tea and fruits; however, the amounts vary considerably. For cocoa, the flavanol content depends on the bean, such as the seed it grows from [6], the manufacturing process, such as heavy alkalisation and temperature of roasting [7], and the final product, (e.g., milk chocolate versus dark chocolate) [8].

Cocoa flavanols have been shown to possess anti-inflammatory and antioxidant effects, with epicatechin the most potent monomer of the flavanol group [9]. Currently, cardiovascular benefits, such as improved flow mediated dilation and reduced blood pressure, have been observed following various doses of CF, such as, 918 mg [10], 701 mg [11], 750 mg [12], and 917 mg [13] and epicatechin intakes as low as 25 mg [14] and 46 mg [15]. Regarding epicatechin, greater efficacy has been reported at higher epicatechin doses (see review [16]). These benefits have been observed following supplementation periods ranging from the same day of testing [11, 13], to seven days [10], and 30 days [12]. Additionally, CF may be beneficial for reducing markers of oxidative stress (defined as an imbalance in the generation of various reactive species and antioxidants [17]) and inflammation [18, 19]. The role of CF in modulating inflammation may stem from their capacity to influence signalling cascades, i.e., via an alteration to eicosanoid production [20], and reducing the activation of certain inflammatory transcription factors, e.g., nuclear factor kappa-beta [21]. Given that exercise-induced muscle damage (EIMD) is thought to partly stem from inflammation and oxidative stress, CF may be able to attenuate functional symptoms that impede athlete recovery, such as muscular soreness and deficits in muscle function [19, 22].

Reactive oxygen species (ROS) are produced as part of normal metabolic processes, such as cellular respiration, and in certain scenarios, such as exercise, ROS are produced in high amounts [2]. Various ROS molecules are involved in a plethora of functions at a cellular level, including, growth and proliferation [23], immune response [24] and apoptosis [25]. Additionally, it is believed that ROS act as signalling molecules in various tissues; however, this is still not fully understood due to the numerous ROS produced at rest and during exercise [26]. Antioxidant defence systems maintain a balance between ROS production and neutralisation; if the production of ROS outweighs their neutralisation, then proteins, lipids and DNA may be oxidised altering their function [27]. This process is typically referred to as oxidative stress. Alternatively, if cells are exposed to low levels of ROS, such as during moderate intensity exercise, they may act as signalling molecules for skeletal muscle adaptations [28]. Such adaptations include an increase in endogenous antioxidants such as superoxide dismutase, glutathione peroxidase and catalase, reduced oxidative damage from exercise and an improved resistance to oxidative stress [29]. The mechanisms by which CF modulate redox metabolism and oxidative stress are not entirely clear, but activation of the nuclear factor erythroid 2-related factor 2 (Nrf2) transcription pathway, which activates a battery of cytoprotective protein with antioxidant and anti-inflammatory functions is a potential candidate [30]. For example, it has been observed that supplementation with catechin results in an increase in the expression of heme-oxygenase 1, an enzyme with antioxidant and anti-inflammatory functions (31), via upregulation of Nrf2 activity [30]. Moreover, cells treated with CF induced an increase in glutathione peroxidase and glutathione reductase, likely via Nrf2 activation [32]. In addition, CF treatment has been shown to prevent a depletion in reduced glutathione and replenish glutathione peroxidase, as well as effectively limiting lipid and protein peroxidation [33]. Collectively, these studies suggest CF may modulate oxidative stress, at least partly via redox sensitive pathways, e.g., stimulating Nrf2 which in turn leads to an increase in redox enzyme expression.

Strenuous exercise may generate large amounts of ROS that leads to oxidative stress. The ROS produced is thought to stem from the increase in cellular respiration, and/or immune cells like neutrophils [1,34]. Leukocytes that accumulate in the muscle after EIMD evoke a respiratory burst, whereby macrophages and neutrophils produce large amounts of ROS to lyse cellular debris and begin regeneration. However, it has been proposed that during this process ROS may also induce lipid peroxidation in nearby healthy tissues [35]. It is thought that this damage to neighbouring cells might contribute to EIMD, and at least partly explain why decrements in muscle function and increased muscle soreness can persist for several days after strenuous exercise [36].

Therefore, the aim of this narrative review was to critically examine research on the effects of CF on oxidative stress, inflammation, muscle function, perceived soreness, and exercise performance. This review builds on previous work by Decroix, Soares 19] that reviewed the effects of CF on exercise performance. The present review includes research completed since the aforementioned article and unlike Decroix and colleagues focuses on CF and EIMD.

To identify the appropriate literature, an in-depth, systematic search of five separate databases was performed (PubMed, Scopus, Web of Science, ScienceOpen and MEDLINE) using specific search terms (‘cocoa flavanols,’ OR ‘dark chocolate,’ AND ‘muscle damage,’ OR ‘muscle recovery,’ OR ‘exercise recovery,’ OR ‘exercise-induced muscle damage,’ OR ‘exercise’). All reviewed studies conducted the investigations in humans.

### Impact of cocoa flavanols on exercise-induced oxidative stress « Table 1»

**Table 1:** The effect of CF supplementation on exercise-induced oxidative stress

Reference	Participants	Nutritional intervention	Supplementation period	Exercise stimulus	Measure(s)	Key outcome(s)
Allgrove et al. [42]	20 healthy males Age 22 ± 4 years Mass 74.6 ± 8 kg *V̇*O_2max_ 53.1 ± 7.0 ml·kg^−1^ Power output 300 ± 30 W	CF: 80 g dark chocolate a day for 14 days, 197.4 mg CF per dose (EPI: 77.4 mg, CAT: 31.2 mg) CON: 56.8 g iso-CHO-fat control chocolate, 0 mg CF	Each day for 14 days, with a half dose 2 h pre-exercise	Cycling at 60% *V̇*O_2max_ for 1.5 h, intensity raised to 90% every 10 min for 30 s 5 min post cycling there was a time to exhaustion trial at 90% *V̇*O_2max_	(i) F_2_-isoprostanes (ii) Oxidised LDLs (iii) Plasma uric acid (iv) TEAC (v) Plasma vitamin C	(i) significantly ↓ in CF group (ii) significantly ↓ across each time point in CF group (iii) ↑ post-exercise in both treatments (iv) ↔ between groups (v) ↔ between groups
Davison et al. [47]	14 healthy males Age 22 ± 1 years Mass 71.6 ± 1.6 kg *V̇*O_2max_ 53.1 ± 1.9 ml·kg^−1^ min^−1^ Power output 300 ± 12 W	CF: 100 g dark chocolate 246.8 mg CF (EPI: 96.8 mg, CAT: 39.1 mg) CON: isomacronutrient control, 0 mg CF None: water	Acute dose 2 h pre-exercise	Cycling at ~ 60% *V̇*O_2max_ for 2.5 h	(i) F_2_-isoprostanes (ii) Plasma Vitamin C (iii) TEAC	(i) ↓ CF group vs CON (ii) ↔ between groups (iii) ↑ pre-exercise CF vs CON
de Carvalho et al. [49]	13 trained males Age 21 ± 2 years Stature 180 ± 0.05 cm Mass 87.02 ± 8.03 kg	CF: CHO + protein cocoa beverage, 306 mg CF per beverage CON: cocoa based CHO + protein beverage, 0 mg CF	7 days, beverage consumed twice daily	Five sets of 20 drop jumps from 0.6 m, 10 s between jumps and 2 min interset rest	Urinary F_2_-isoprostanes	↔ between groups
Decroix et al. [43]	12 well-trained males Age 30 ± 3 years Stature 177.9 ± 8.8 cm Mass 72.8 ± 7.8 kg *V̇*O_2max_ 63.0 ± 3.5 ml·kg^−1^ min^−1^	CF: cocoa drink, 900 mg CF (EPI: 185 mg, CAT: 20 mg) CON: placebo, 15 mg CF (EPI: 0 mg, CAT: 0 mg)	Acute dose 1.5 pre-exercise	Two 30 min time trials 60 min apart, performed at a ~ 75% peak power output	(i) Uric acid (ii) MDA (iii) TEAC	(i) ↑ in CF vs CON (ii) ↔ between group (iii) ↑ in CF vs CON
Decroix et al. [45]	14 well-trained males Age 31 ± 3 years Stature 180 ± 5 cm Mass 73 ± 7 kg *V̇*O_2max_ 62.9 ± 5.8 ml·kg^−1^ min^−1^ Peak Power Output 366 ± 45 W	CF: Capsule, 530 mg CF (EPI: 100 mg, CAT: 21 mg) CON: 1,764 mg maltodextrin	Consumed daily for six days and then a seventh on the day of testing	20 min steady state cycling at 45% peak power output 20 min time trial beginning at 75% peak power output Completed in normoxic and hypoxic environments	(i) TEAC (ii) Uric acid (iii) MDA	(i) ↔ (ii) ↔ (iii) CF blunted ↑ in both N and H
Fraga et al. [41]	28 trained males Age 18 ± 1 years Mass 74 ± 1 kg	CF: 105 g chocolate confectionery, 168 mg CF (EPI + CAT: 39 mg) CON: 105 g cocoa butter chocolate, < 5 mg CF	Sub-chronic, 14 day consumption	Soccer training sessions twice per week and one match per week	(i) MDA (ii) Urate (iii) Oxo^8^dG (iv) TRAP (v) α-tocopherol (vi) lycopene vii) β-carotene (viii) coenzyme Q-10	(i) Post CF ↓ by 12% CON ↑ by 10% (ii) ↓ by 11% in CF (iii-viii) ↔ between groups
Morgan et al. [48]	10 active males Age 23 ± 3 years Stature 184 ± 59 cm Mass 85.3 ± 12.0 kg Single leg 1RM 90.4 ± 19.0 kg	CF: 330 ml cacao juice, 74 mg CF (EPI: 8 mg, CAT: 43 mg) CON: 330 ml CHO and flavour matched placebo, 0 mg CF	10 days supplementation (7 days pre-exercise, 3 days post)	10 sets of 10 single leg knee extensions at ~ 80% 1RM	Protein carbonylation	Protein carbonylation not elevated following exercise protocol
Taub et al. [50]	17 sedentary (9 males 8 females) participants CF: Age 50 ± 3 Stature 168 ± 3 Mass 78.8 ± 5.6 *V̇*O_2max_ 22.9 ± 1.9 ml·kg^−1^ min^−1^ CON: Age 50 ± 2 Stature 175 ± 5 Mass 92.2 ± 9.7 *V̇*O_2max_ 24 ± 1.7 ml·kg^−1^ min^−1^	CF: 20 g dark chocolate, 175.2 mg CF (EPI: 26 mg, CAT: 4.6) CON: 20 g placebo chocolate	Chronic (3 months daily intake)	Cycling exercise including *V̇*O_2max_	(i) GSH:GSSG ratio (ii) Protein carbonylation	(i) significant ↑ in CF group vs CON (ii) significant ↓ in CF group vs CON
Wiswedel et al. [44]	20 untrained males Age ~ 20–25	CF: cocoa drink, 185 mg CF CON: cocoa drink 14 mg CF	Acute, 2 h pre cycling exercise	Cycling at 75 W increasing to 150 W for 10 min	(i) F2-isoprostanes (ii) MDA (iii) α-tocopherol (iv) ascorbate (v) TAC	(i) CON small ↑ 2 and 4 h post-consumption, CF did not Significant difference CF vs CON 2 and 4 h post-intake following exercise (ii–v) ↔ between groups

CF, cocoa flavanols; EPI, epicatechin; CAT, catechin; CON, control;CHO, carbohydrate; TEAC, Trolox equivalent antioxidant capacity; MDA, malonaldehyde; LDL, low density lipoprotein; Oxo^8^dG, 8-Oxo-2’-deoxyguanosine; 1RM, one rep max; GSH:GSSG, reduced glutathione: oxidised glutathione ratio; TAC, total antioxidant capacity; TRAP, total relative antioxidant potency; ↑, increase; ↓, decrease; ↔ , no significant effect/change

Antioxidants maintain redox status by neutralising ROS produced by metabolic reactions [37]. However, as explained in the introduction, the upregulation of ROS can lead to oxidative stress if cellular antioxidant capacity is overwhelmed. Oxidative stress in skeletal muscle decreases force output [38], likely through a reduction in calcium (Ca^2+^) sensitivity in the myofibrils and reduced activity of calcium ATPase, suggesting contractile dysfunction partly due to the accumulation of Ca^2+^ [39,40]. Therefore, an increase in antioxidant capacity may lead to improvements in performance and recovery through reductions in fatigue associated with ROS during and after exercise.

Two studies that examined the effects of CF on markers of oxidative stress observed significant interaction effects following a 14-day sub-chronic supplementation period [41,42]. Allgrove and colleagues observed that F_2_-isoprostanes and oxidised low density lipoprotein (markers of oxidative stress) were significantly lower in the treatment group, supplementing 197.4 mg CF and 77.4 mg epicatechin, versus placebo post 90 min of cycling at 60% *V̇*O_2max_, interspersed with 30 s efforts at 90% *V̇*O_2max_ every 10 min [42]. Similarly, Fraga and colleagues [29]) found that regular CF intake (168 mg) alongside soccer training and match play over a 14-day period resulted in a 12% decrease in malondialdehyde (MDA; a marker of lipid peroxidation), whereas in the placebo condition values increased by 10%, indicating a reduction in oxidative stress associated with training and match play. A study by Decroix, Tonoli [43] observed that although cycling time trial exercise increased MDA concentrations, CF had no significant impact compared to placebo. Wiswedel, Hirsch [44] also found no significant treatment effect of CF on MDA concentrations following cycling exercise. Interestingly, Wiswedel, Hirsch [44] included a no exercise control and found that the high flavanol group had a lesser increase in MDA than the low CF group four- and six-hours post-ingestion. In contrast, when supplementing 1765 mg of cocoa extract (containing 530 mg CF) for six days in the lead up to exercise and once more immediately before, CF blunted the exercise-induced rise in MDA concentrations [45]. These changes imply that sub-chronic consumption of CF may reduce exercise-induced oxidative stress more effectively than an acute dose. The results suggest that CF may be a potent antioxidant, with plasma MDA levels decreasing from baseline over a 14-day period of 168 mg of CF consumption a day [41]. These findings may have applicability to clinical populations as it has been reported previously that CF supplementation prevents systemic oxidative stress (measured via plasma MDA and urinary prostaglandin F2α) in type II diabetes and cancer [46]. Notwithstanding, the other markers of oxidative stress and antioxidant activity were not affected by the treatment (8-oxo-2-deoxyguanosine and total relative antioxidant potency respectively), with a possible explanation being the relatively low amount of collective epicatechin and catechin in the treatment—only 39 mg per dose [41], or the markers were not sensitive enough to detect changes in soccer players.

Where Allgrove and colleagues found a significant difference for F_2_-isoprostanes post-cycling exercise after a sub-chronic dosing protocol of CF, both Davison, Callister [47] (246.8 mg, 96.8 mg epicatechin) and Wiswedel, Hirsch [44] (187 mg) observed that even an acute dose of CF pre-cycling exercise elicited reductions in F_2_-isoprostanes when compared to placebo in a crossover design. These were the only acute dose studies to observe any treatment effect on oxidative stress as the other two reported no differences between treatments [48,49]. The only study to assess oxidative stress over a chronic supplementation period had participants consuming 175.2 mg daily for 30 days and found that CF significantly increased the reduced glutathione/oxidised glutathione ratio and reduced protein carbonylation [50]. This again indicates that prolonged supplementation may be more beneficial than solely acute consumption.

Data regarding uric acid/urate is conflicting across studies. Decroix, Tonoli [43] reported that an acute dose of 900 mg CF increased uric acid following two 30 min time trials. In contrast, Fraga, Actis-Goretta [41] found that sub-chronic dosing of 168 mg CF per day decreased urate levels by 11% compared to the beginning of supplementation, Decroix, Tonoli [45] also found that 1765 mg cocoa extract (530 mg CF) per day over a seven day period did not influence uric acid concentrations at rest or post-exercise. However, the contrasting observations may be attributed to the fact that Fraga, Actis-Goretta [41] collected blood samples on a rest day, while Decroix, Tonoli [43] took blood samples immediately post-exercise; which has been observed to increase uric acid concentrations 1–2 h post intense exercise [51]. As Decroix, Tonoli [45] took samples at rest and post-exercise whilst using the highest dose of CF and found no impact, this may imply that the mechanism that CF act as an antioxidant may be independent to the mechanism behind changes in uric acid concentrations. Uric acid can be used as a marker of oxidative stress due to its role in the conversion of xanthine dehydrogenase to xanthine oxidase, which then increases the production of ROS [52]. Counterintuitively, uric acid is also one of the predominant antioxidants found within the plasma [53,54]. The role of uric acid as a pro-oxidant within the cellular compartment, coupled with its role as an antioxidant in the plasma, make it difficult to draw practical conclusions from antioxidant based nutritional studies. Additionally, certain flavonoids, such as quercetin due to its chemical structure, may act as an inhibitor of the production of xanthine oxidase (an enzyme that increases ROS concentrations) and as such have a direct influence on uric acid concentrations [55].

However, there are times during an athletes’ training when reducing oxidative stress may not be desired, such as during pre-season when adaptations from exercise are the priority as opposed to accelerated recovery. The adaptations associated with oxidative stress during and following exercise include improved cellular repair systems and reduced production of damaging ROS [56]. However, these exercise related training adaptations may be hindered by regular high doses of antioxidant compounds and prevent or obstruct key cellular functions associated with ROS [57]. Nevertheless, a recent meta-analysis identified that the evidence for a blunting effect of (poly)phenol supplementation on exercise adaptations is equivocal, more research is needed to fully understand how (poly)phenols may augment exercise adaptations [58].

### Impact of cocoa flavanols on exercise-induced inflammation Table 2

**Table 2:** The effect of CF supplementation on exercise-induced inflammation

Reference	Participants	Nutritional Intervention	Supplementation period	Exercise stimulus	Measure(s)	Key outcome(s)
Allgrove et al. [42]	20 healthy males Age 22 ± 4 years Mass 74.6 ± 8 kg *V̇*O_2max_ 53.1 ± 7.0 ml·kg^−1^ Power output 300 ± 30 W	CF: 80 g dark chocolate a day for 14 days, 197.4 mg CF per dose (EPI: 77.4 mg, CAT: 31.2 mg) CON: 56.8 g iso-CHO-fat control chocolate, 0 mg CF	Each day for 14 days, with a half dose 2 h pre-exercise	Cycling at 60% *V̇*O_2max_ for 1.5 h, intensity raised to 90% every 10 min for 30 s 5 min post cycling there was a time to exhaustion trial at 90% *V̇*O_2max_	(i) Circulating leukocytes (ii) Neutrophils (iii) IL-10 (iv) IL-6 (v) IL-1ra	(i–v) ↔ between groups
Davison et al. [47]	14 healthy males Age 22 ± 1 years Mass 71.6 ± 1.6 kg *V̇*O_2max_ 53.1 ± 1.9 ml·kg^−1^ min^−1^ Power output 300 ± 12 W	CF: 100 g dark chocolate 246.8 mg CF (EPI: 96.8 mg, CAT: 39.1 mg) CON: isomacronutrient control, 0 mg CF None: water	Acute dose 2 h pre-exercise	Cycling at ~ 60% *V̇*O_2max_ for 2.5 h	IL-6	↔ between groups
Decroix et al. [43]	12 well-trained males Age 30 ± 3 years Stature 177.9 ± 8.8 cm Mass 72.8 ± 7.8 kg *V̇*O_2max_ 63.0 ± 3.5 ml·kg^−1^ min^−1^	CF: cocoa drink, 900 mg CF (EPI: 185 mg, CAT: 20 mg) CON: placebo, 15 mg CF (EPI: 0 mg, CAT: 0 mg)	Acute dose 1.5 pre-exercise	Two 30 min time trials 60 min apart, performed at a ~ 75% peak power output	(i) TNF-α (ii) IL-1 (iii) IL-6	(i) ↔ between groups (ii) ↔ between groups
Morgan et al. [48]	10 active males Age 23 ± 3 years Stature 184 ± 59 cm Mass 85.3 ± 12.0 kg Single leg 1RM 90.4 ± 19.0 kg	CF: 330 ml cacao juice, 74 mg CF (EPI: 8 mg, CAT: 43 mg) CON: 330 ml CHO and flavour matched placebo, 0 mg CF	10 days supplementation (7 days pre-exercise, 3 days post)	10 sets of 10 single leg knee extensions at ~ 80% 1RM	(i) CRP (ii) IL-6	(i) ↔ between groups (ii) ↔ between groups
Taub et al. [50]	17 sedentary (9 males 8 females) participants CF: Age 50 ± 3 Stature 168 ± 3 Mass 78.8 ± 5.6 *V̇*O_2max_ 22.9 ± 1.9 ml·kg^−1^ min^−1^ CON: Age 50 ± 2 Stature 175 ± 5 Mass 92.2 ± 9.7 *V̇*O_2max_ 24 ± 1.7 ml·kg^−1^ min^−1^	CF: 20 g dark chocolate, 175.2 mg CF (EPI: 26 mg, CAT: 4.6) CON: 20 g placebo chocolate	Chronic (3 months daily intake)	Cycling exercise including *V̇*O_2max_	CRP	↔ between groups

CF, cocoa flavanols; EPI, epicatechin; CAT, catechin; CON, control; CHO, carbohydrate; IL, interleukin; CRP, c-reactive protein; TNF-α, Tumour-necrosis factor-; ↔ , no significant effect/change

Strenuous exercise resulting in muscle damage evokes an acute inflammatory response [59]. Several studies have observed systemic increases in markers such as interleukin-6 (IL-6), c-reactive protein (CRP) and tumour necrosis factor-α (TNF-α) [60,61] following intense exercise. These markers are typically increased for several hours following exercise, but may persist for several days depending on the severity of the damage [62]. Inflammation, particularly the increase in neutrophils, has been associated with muscle function loss following exercise, suggesting the acute inflammatory response plays a role in recovery after exercise [63].

In vitro studies have shown that CF have anti-inflammatory properties and can reduce tumour necrosis factor-α (TNF-α) from inducing an upregulation of vascular endothelial growth factor activity [64] and inhibit nuclear factor-kappa beta activation [65]. In humans, CF supplementation has been shown to decrease Interleukin-1β and Interleukin-10 levels [66], four weeks of dark chocolate consumption reduced leukocyte accumulation, soluble adhesion molecules, and the expression of adhesion markers on leukocytes [67] (see the recent review by Goya, Martín [68] for more detail). This may indicate that dark chocolate or cocoa powder with a high proportion of CF would perhaps be viable as a therapeutic, anti-inflammatory intervention.

Studies by Allgrove, Farrell [42] and Davison, Callister [47] found that prolonged cycling at 60% $\dot{V}$O_2max_ increased inflammatory markers (IL-6, IL-10 and IL-1ra and IL-6, blood leucocyte count and neutrophil count, respectively) but found no difference between CF supplementation or placebo. Decroix, Tonoli [43] used two 30 min time trials separated by 90 min; the first time trial starting 100 min post ingestion of a 900 mg CF beverage. This resulted in no treatment or time effect on inflammatory markers (TNF-α, IL-1 and IL-6), perhaps implying the stimulus was not intense enough to induce inflammation in a cohort of well-trained cyclists. However, as both Allgrove, Farrell [42] and Davison, Callister [47] used relatively low doses of CF (197.4 mg and 246.8 mg respectively), a higher dose of both total flavanols and epicatechin is perhaps necessary to evoke the purported anti-inflammatory effects of CF [69], in situations that induce an increase in inflammatory markers. These effects include the modulation of particular aspects of the inflammatory cascade, such as, inhibiting platelet aggregation [70] and altering cytokine production via stimulation or inhibition of certain interleukins and growth factors (Selmi, Mao [71] for a review). Therefore, it is possible that for CF to confer anti-inflammatory benefits, the inflammation must be pronounced and/or prolonged. Furthermore, cycling exercise does not include a significant eccentric action; the type of contraction that is most associated with EIMD and as a result may not cause systemic inflammation to reach the same level of studies that involve eccentric biased exercise [72].

Currently, the only EIMD study with CF that measured inflammation was by Morgan, Wollman [48], in this study no differences between treatment groups for IL-6 or CRP, following 100 maximal leg extensions with an elongated eccentric phase (three seconds). However, the researchers utilised a low dose (74 mg) of CF which is potentially why no effect was observed. The lack of studies showing robust changes in inflammation following exercise suggests that the anti-inflammatory effects of CF seen in in vitro studies may not translate to the in vivo environment. It is pertinent that future research investigates the impact of CF on markers of inflammation following EIMD (e.g., TNF-α), potentially including muscle biopsies to provide measured changes of inflammation in the muscle. It should be noted that the inflammatory process is necessary for skeletal muscle adaptation, and by blunting the initial pro-inflammatory phase, it is possible that the muscle regenerative phase can be impaired [73]. Indeed, an adaptation to exercise is the increased activity of peroxisome proliferator-activated receptor γ co-activator 1α, which may aid the phenotype switch of macrophages from pro- to anti-inflammatory and reduce the expression of genes associated with oxidative stress [74,75]. Therefore, forgoing an anti-inflammatory intervention may be effective when adaptations to exercise are the priority, akin to adaptations related to ROS and oxidative stress. However, the evidence that long term supplementation of CF, (poly)phenols, or other antioxidant supplements (e.g., vitamin C and E) can inhibit training adaptations is equivocal [76–78]; as such, more research is warranted to better understand how these compounds may influence exercise adaptations.

### Impact of cocoa flavanols on the recovery of muscle function « Table 3»

**Table 3:** The effect of CF supplementation on exercise-induced changes in muscle function

Reference	Participants	Nutritional Intervention	Supplementation period	Exercise stimulus	Measure(s)	Key outcome(s)
Corr, Field [80]	23 active (13 females, 10 males) participants Age 24 ± 5 years Stature 170 ± 9 cm Mass 69 ± 12 kg	CF: CHO + protein cocoa beverage, 1245 mg (EPI: 150 mg) OR 830 mg (EPI: 99 mg) CON: CHO + protein	Acute, immediately post-exercise	Five sets of 10 maximal concentric-eccentric contractions of the knee flexors on an isokinetic dynamometer at 60°/s	MVC at 30° and 60°	↔ between groups, large effect sizes observed 1245 vs CON and 830 vs CON
de Carvalho, Fisher [49]	13 trained males Age 21 ± 2 years Stature 180 ± 0.05 cm Mass 87.02 ± 8.03 kg	CF: CHO + protein cocoa beverage, 306 mg CF per beverage CON: cocoa based CHO + protein beverage, 0 mg CF	7 days, beverage consumed twice daily	Five sets of 20 drop jumps from 0.6 m, 10 s between jumps and 2 min interset rest	(i) MVC (ii) Vertical jump	(i) ↔ between groups (ii) ↔ between groups
Morgan, Wollman [48]	10 active males Age 23 ± 3 years Stature 184 ± 59 cm Mass 85.3 ± 12.0 kg Single leg 1RM 90.4 ± 19.0 kg	CF: 330 ml cacao juice, 74 mg CF (EPI: 8 mg, CAT: 43 mg) CON: 330 ml CHO and flavour matched placebo, 0 mg CF	10 days supplementation (7 days pre-exercise, 3 days post)	10 sets of 10 single leg knee extensions at ~ 80% 1RM	(i) MVC (ii) CMJ	(i) ↔ between groups (ii) ↑ recovery of CMJ
Peschek, Pritchett [79]	8 well-trained males Age 25 ± 6 years Stature 182.1 ± 6.3 cm Mass 73.4 ± 7.0 *V̇*O_2max_ 64.4 ± 7.6 ml·kg^−1^ min^−1^	CF: Cocoa based CHO protein beverage with added cocoa, 350 mg CF CON: cocoa based CHO protein beverage, 0 mg CF	Acute ingestion of two beverages separated by 2 h post-exercise protocol	30 min downhill running at a − 10% gradient at 70% *V̇*O_2max_	MVC	↔ between groups

CF, cocoa flavanols; EPI, epicatechin; CAT, catechin; CON, control; CHO, carbohydrate; 1RM, one rep max; MVC, maximal voluntary contraction; CMJ, countermovement jump; ↑, increase; ↔ , no significant effect/change

Muscle function is negatively impacted by EIMD, with reductions in muscle force and power capacity evident for several days following strenuous exercise. However, based on the current evidence it seems that CF supplementation has minimal, if not any, impact on maximal voluntary contraction (MVC; as measured using peak torque) with no effect observed on knee extensor [48,49,79] or knee flexor strength recovery [80]. Interestingly however, Corr, Field [80] found that at 24 and 48 h post-exercise there were large effect sizes following an acute high dose of 1245 mg CF compared to the control for MVC at 30 degrees and MVC percentage change at 60 and 30 degrees, although no significant differences were observed. Therefore, it is feasible that the dose of 1245 mg CF has the potential to be effective but may require repeated dosing throughout the recovery period instead of a singular acute dose to be truly efficacious. Currently, for (poly)phenols it has been suggested that > three days of supplementation above 1000 mg may be required to observe an ergogenic benefit [81], however no CF research has been performed fulfilling that criteria, with only one study using a dose above 1000 mg of CF [80].

It is noteworthy that only Morgan, Wollman [42] and Corr, Field [80] observed notable muscle damage based on decrements in muscle function across groups [82]. To best understand the mechanisms behind CFs role in muscle damage recovery, it would be prudent to ensure symptoms of EIMD such as a decrease in muscle function are pronounced. In fact, it is noteworthy that the participants in de Carvalho, Fisher [49] had fully recovered muscle function (based on peak torque data) 48 h post-exercise, indicating that the 100 drop-jump protocol did not elicit significant damage in a group of elite rugby players. Therefore, in populations with high baseline strength and power, protocols designed to induce EIMD need to be of a sufficient magnitude. Similarly, Peschek, Pritchett [79] observed 2–5% decrements in the control group and 10–22% in the CF group from pre to 24 h post, indicating that perhaps CF ingestion exacerbated muscle damage or only the CF group suffered the deleterious effects of the EIMD protocol. Interestingly, from 24 to 48 h post-exercise the CF groups muscle function improved, whereas no changes occurred in the control group. Nevertheless, as the control group did not experience pronounced levels of muscle damage, it is possible that the protocol was not sufficient to adequately study the effects of CF on muscle function. Nevertheless, if the protocol is not representative of the training loads regularly experienced by those individuals, the functional relevance of investigating EIMD and CF supplementation becomes questionable. A further measure of muscle function used was vertical jump height, in which they found no significant differences between groups [49].

In contrast, Morgan, Wollman [48] found that an acute dose of CF (74 mg) aided countermovement jump (CMJ) height recovery as participants returned to 95% of baseline at 48 h in the CF group compared to 87% in the placebo group. However, in this study they consumed a much lower dose than used previously in the literature, especially the epicatechin content (8 mg). Furthermore, the researchers utilised a unilateral EIMD protocol yet the CMJ is a bilateral test, which could have influenced the findings. Instead, a more appropriate test could have been implemented, e.g., a single leg CMJ, as differences between participants’ dominant and non-dominant legs may have been a confounding variable for jump height.

### Impact of cocoa flavanols on perceived soreness «Table 4»

**Table 4:** The effect of CF supplementation on exercise-induced changes in perceived soreness

Reference	Participants	Nutritional Intervention	Supplementation period			Exercise stimulus	Measure(s)	Key outcome(s)
Corr, Field [80]	23 active (13 females, 10 males) participants Age 24 ± 5 years Stature 170 ± 9 cm Mass 69 ± 12 kg	CF: CHO + protein cocoa beverage, 1245 mg (EPI: 150 mg) OR 830 mg (EPI: 99 mg) CON: CHO + protein		Acute, immediately post-exercise	Five sets of 10 maximal concentric-eccentric contractions of the knee flexors on an isokinetic dynamometer at 60°/s		(i) VAS (ii) LEFS	(i) ↔ between groups, large effect size 1245 vs CON (ii) ↔ between groups
de Carvalho et al. [49]	13 trained males Age 21 ± 2 years Stature 180 ± 0.05 cm Mass 87.02 ± 8.03 kg	CF: CHO + protein cocoa beverage, 306 mg CF per beverage CON: cocoa based CHO + protein beverage, 0 mg CF		7 days, beverage consumed twice daily	Five sets of 20 drop jumps from 0.6 m, 10 s between jumps and 2 min interset rest		(i) VAS (ii) LEFS	(i) ↔ between groups (ii) ↔ between groups
Morgan et al. [48]	10 active males Age 23 ± 3 years Stature 184 ± 59 cm Mass 85.3 ± 12.0 kg Single leg 1RM 90.4 ± 19.0 kg	CF: 330 ml cacao juice, 74 mg CF (EPI: 8 mg, CAT: 43 mg) CON: 330 ml CHO and flavour matched placebo, 0 mg CF		10 days supplementation (7 days pre-exercise, 3 days post)	10 sets of 10 single leg knee extensions at ~ 80% 1RM		VAS	↔ between groups
Peschek et al. [79]	8 well-trained males Age 25 ± 6 years Stature 182.1 ± 6.3 cm Mass 73.4 ± 7.0 *V̇*O_2max_ 64.4 ± 7.6 ml·kg^−1^ min^−1^	CF: Cocoa based CHO protein beverage with added cocoa, 350 mg CF CON: cocoa based CHO protein beverage, 0 mg CF		Acute ingestion of two beverages separated by 2 h post-exercise protocol	30 min downhill running at a − 10% gradient at 70% *V̇*O_2max_		(i) VAS (ii) LEFS	(i) ↔ between groups (ii) ↔ between groups

CF, cocoa flavanols; EPI, epicatechin; CAT, catechin; CON, control; CHO, carbohydrate; VAS, visual analogue scale; LEFS, lower extremity functional scale; 1RM, one rep max; ↔ , no significant effect/change

Measures of perceived soreness are predominantly subjective in nature, typically measured using validated scales to quantify subjective pain, soreness and discomfort such as a visual analogue scale (VAS) [83] or lower extremity functional scale (LEFS) [84]. As muscular soreness is ubiquitous with EIMD, most studies investigating muscle damage utilised these measures of perceived soreness as a way of tracking recovery [48,49,79,80]. Peschek, Pritchett [79] administered two doses of 350 mg CF post EIMD which were separated by two hours and found no effect of treatment on VAS or LEFS scores. Interestingly, the increase in soreness from baseline to 24 and 48 h post was not significant. This suggests that the protocol used (downhill running at a −10% gradient for 30 min) may not have induced significant levels of muscle damage in a cohort of well-trained endurance athletes.

Similarly, de Carvalho, Fisher [49] did not find any interaction effect of the treatment following the EIMD protocol with only minor changes from baseline at 48 h, even though this is when DOMS is known to peak [60]. Out of the four studies only Morgan, Wollman [48] and Corr et al. [54] found a main effect of time on VAS scores following their respective protocols (100 knee extensions and 100 isokinetic hamstring curls respectively). Neither found a significant difference between conditions; although as mentioned previously Morgan, Wollman [31] used a small dose of 74 mg CF and a very low dose of 8 mg epicatechin. This amount is unlikely to exert any benefit as the required amounts to have a physiological influence are reported to begin around 400–700 mg [13] and at an epicatechin intake of 50 mg [14]. However, Corr and colleagues [54] did find a large effect size for VAS scores with a greater dose of 1245 mg CF when compared to the control at 48 h.

### Impact of cocoa flavanols on exercise performance «Table 5»

**Table 5:** The effect of CF supplementation on exercise performance

Reference	Participants	Nutritional Intervention	Supplementation period	Exercise stimulus	Measure(s)	Key outcome(s)
de Carvalho, Fisher [49]	13 trained males Age 21 ± 2 years Stature 180 ± 0.05 cm Mass 87.02 ± 8.03 kg	CF: CHO + protein cocoa beverage, 306 mg CF per beverage CON: cocoa based CHO + protein beverage, 0 mg CF	7 days, beverage consumed twice daily	Five sets of 20 drop jumps from 0.6 m, 10 s between jumps and 2 min interset rest	Yo-Yo intermittent test	* ↔ *between groups CF group ↑ 9.85% compared to baseline CON ↓ 5.8% compared to baseline
Decroix, Tonoli [43]	12 well-trained males Age 30 ± 3 years Stature 177.9 ± 8.8 cm Mass 72.8 ± 7.8 kg *V̇*O_2max_ 63.0 ± 3.5 ml·kg^−1^ min^−1^	CF: cocoa drink, 900 mg CF (EPI: 185 mg, CAT: 20 mg) CON: placebo, 15 mg CF (EPI: 0 mg, CAT: 0 mg)	Acute dose 1.5 pre-exercise	Two 30 min time trials 60 min apart, performed at a ~ 75% peak power output	(i) Time trial (ii) PPO	(i) ↔ between groups (ii) PPO ↑ after 25 min in the 1st time trial for CF
Decroix et al. [45]	14 well-trained males Age 31 ± 3 years Stature 180 ± 5 cm Mass 73 ± 7 kg *V̇*O_2max_ 62.9 ± 5.8 ml·kg^−1^ min^−1^ Peak Power Output 366 ± 45 W	CF: Capsule, 530 mg CF (EPI: 100 mg, CAT: 21 mg) CON: 1,764 mg maltodextrin	Consumed daily for six days and then a seventh on the day of testing	20 min steady state cycling at 45% peak power output 20 min time trial beginning at 75% peak power output Completed in normoxic and hypoxic environments	Time trial	* ↔ *between groups
Fraga, Actis-Goretta [41]	28 trained males Age 18 ± 1 years Mass 74 ± 1 kg	CF: 105 g chocolate confectionery, 168 mg CF (EPI + CAT: 39 mg) CON: 105 g cocoa butter chocolate, < 5 mg CF	Sub-chronic, 14 day consumption	Soccer training sessions twice per week and one match per week	*V̇*O_2max_ shuttle run	* ↔ *between groups
Patel, Brouner [85]	9 trained males Age 21 ± 1 years Stature 177 ± 9.4 cm Mass 76.0 ± 9.3 kg *V̇*O_2max_ 41.89 ± 5.4 ml·kg^−1^ min^−1^	CF: 40 g dark chocolate, 259 mg CF CON: 40 g white chocolate	Sub-chronic, 14 days consumption	20 min cycling at 80% of gas exchange threshold followed by a 2 min maximal sprint time trial	Time trial	A 17% ↑ in distance covered was observed following CF supplementation
Patel, Brouner [86]	15 healthy (10 males, 5 females) participants Age 30 ± 7 years Stature 176.8 ± 8.6 cm Mass 80.3 ± 8.4 kg *V̇*O_2max_ Males: 51.1 ± 3.5 Females: 41.6 ± 5.5 ml·kg^−1^ min^−1^	CF: dark chocolate, 1060 mg CF, 764 mg CF, or 406 mg CF CON: 88 mg CF	Acute ingestion, 2 h pre-exercise	2-min incremental cycling warm-up until 80% of GET then maintained for 40 min. Followed by an incremental test to failure	(i) *V̇*O_2_ (ii) PPO	(i) *↔ *between treatment (ii)* ↔ *between treatment
Peschek, Pritchett [79]	8 well-trained males Age 25 ± 6 years Stature 182.1 ± 6.3 cm Mass 73.4 ± 7.0 *V̇*O_2max_ 64.4 ± 7.6 ml·kg^−1^ min^−1^	CF: Cocoa based CHO protein beverage with added cocoa, 350 mg CF CON: cocoa based CHO protein beverage, 0 mg CF	Acute ingestion of two beverages separated by 2 h post-exercise protocol	30 min downhill running at a − 10% gradient at 70% *V̇*O_2max_	5 km time trial	* ↔ *between treatments
Sadler, Draijer [89]	17 healthy (11 males, 6 females) participants Age 45 ± 6 years Stature 162 ± 0.1 cm Mass 68.2 ± 17.7 kg	CF: capsule containing 100 mg CF (EPI + CAT: 22 mg)	Four capsules taken daily (two in the morning and two in the evening) for seven consecutive days Four capsules consumed 45 min prior to arrival at the lab on the day of the protocol (7^th^ day)	6 min cycling at 80% GET threshold × 3 and 1 bout of cycling at 60% of the difference between GET and *V̇*O_2peak_ until exhaustion	(i) t*V̇*O_2_ (ii) ET	(i) 15% ↓ in CF group than CON (ii) ↔ between treatments for ET
Stellingwerff, Godin [88]	16 healthy males Age 30 ± 6 years Stature 179.9 ± 7.8 cm Mass 72.8 ± 6.0 kg *V̇*O_2peak_ 56.3 ± 5.7 ml·kg^−1^ min^−1^	CF: 561 kcal dark chocolate, 240 mg CF (EPI: 89 mg, CAT: 24 mg) CON: chocolate ~ 0 mg CF	Acute ingestion 2 h pre-exercise	Cycled for 2.5 h at ~ 45% *V̇*O_2max_, followed by 15 min time trial	Time trial	* ↔ *between treatments
Taub et al. [50]	17 sedentary (9 males 8 females) participants CF: Age 50 ± 3 Stature 168 ± 3 Mass 78.8 ± 5.6 *V̇*O_2max_ 22.9 ± 1.9 ml·kg^−1^ min^−1^ CON: Age 50 ± 2 Stature 175 ± 5 Mass 92.2 ± 9.7 *V̇*O_2max_ 24 ± 1.7 ml·kg^−1^ min^−1^	CF: 20 g dark chocolate, 175.2 mg CF (EPI: 26 mg, CAT: 4.6) CON: 20 g placebo chocolate	Chronic (3 months daily intake)	Cycling exercise including *V̇*O_2max_	(i) *V̇*O_2max_ (ii) Power	(i) Significant ↑ in CF vs CON (ii) CF significant ↑, CON ↔

CF, cocoa flavanols; EPI, epicatechin; CAT, catechin; CON, control; CHO, carbohydrate; PPO, peak power output; tV̇O_2_, time constant of the fundamental phase of V̇O_2_ kinetics; ET, exercise tolerance; ↑, increase; ↓, decrease; ↔ , no significant effect/change

The impact that CF may have on performance is likely through the antioxidant potential of the cocoa and delayed ROS-induced fatigue. Patel, Brouner [85] measured performance using maximal distance timed sprint trial, which was completed after 20 min of cycling. It was found that 259 mg CF consumed daily for 14 days resulted in participants covering 17% more distance than baseline and 13% more distance than a white chocolate control. The mechanism for this increase may be due to CF decreasing ROS production and thereby attenuating fatigue [41,42]. An acute dosing strategy with higher flavanol products did not elicit any cycling performance benefit, only inducing slightly higher nitric oxide levels during exercise, which could aid muscle blood flow [86].

Many sports have limited recovery time between competitions. For example, in field hockey tournaments, matches are often played 48 h apart; similar recovery times are evident in soccer and handball. As a result, it may be pertinent to accelerate recovery and attenuate symptoms of EIMD in these sports [87]. In one study, supplementation of CF (616 mg CF for 7 days) increased distance covered during the Yo-Yo Intermittent test 1 of 9.85% from baseline to 48 h post a 100 drop jump EIMD protocol. Whereas the placebo group covered 5.8% less distance [49]. In this study CF may have reduced any potential oxidative stress that would be associated with training, exercise or the EIMD protocol, which may subsequently delay fatigue.

Even though CF supplementation may improve distance covered in a set amount of time, it may not improve performance related to completing a set amount of work or distance in a time trial setting. Decroix, Tonoli [43], Decroix, Tonoli [45] and Stellingwerff, Godin [88] found no significant differences between groups (CF vs placebo) for time trial performance. However, Decroix and colleagues [37] observed that in a crossover design the CF group tended to complete the first of the two time trials faster (29:47 min placebo vs 29:13 min cocoa), although statistical significance was not reached. It is difficult to ascertain whether the 34 s difference between groups is meaningful, as the trial involved participants completing a set amount of work equivalent to cycling at 75% peak power output for 30 min as fast as possible. As each time trial would have been individualised to each participant any practical conclusions are difficult to make other than that CF may have allowed participants to maintain a slightly higher power output than a placebo [43]. The CF group also produced a higher power output after 25 min (for the final ~ 5 min of the first time trial) compared to placebo (PLA 73.09% vs CF 76.75% of maximal power output). Decroix, Tonoli [45] found no differences for rating of perceived exertion, heart rate, lactate or work performed (kilojoules) within the 20-min time trial between groups in normoxic or hypoxic environments. Interestingly, Stellingwerff, Godin [88] found that performance increased for seven participants following CF supplementation whereas another seven had improved performance following ingestion of the placebo. This may suggest that some individuals are potential ‘non-responders’ to CF supplementation, or that the differences seen were due to chance and not the allocated treatments. Other studies that investigated performance and CF supplementation found no significant differences between groups for 5 km time trial performance or *V̇*O_2max_ [41,79,86]. However, recent work by Sadler, Draijer [89] suggests that 400 mg daily CF supplementation for seven days improves oxygen uptake during moderate-intensity exercise, but this benefit was not observed during high-intensity exercise. Additionally, after three-months of supplementing 175.2 mg/day of CF, Taub, Ramirez-Sanchez [50] observed an increase in participants’ *V̇*O_2max_ by 2.8 ± 1.2 ml kg^−1^ min^−1^ and power values (140.7 ± 11.6 to 148.3 ± 11 watts), whereas there were no significant differences in the placebo group.

### Practical recommendations and future research

The available data suggests it may be beneficial to ingest a moderate dose of CF pre-exercise, with benefits effects on oxidative stress observed at doses ~ 200 mg acutely and if taken more longer term in the lead up to exercise. Higher doses of polyphenols may elicit greater physiological effects in vivo [81] and for CF dosage the amount of epicatechin is an important factor when considering supplementation (≥ 50 mg).

To maximise absorption and bioavailability, CF can be ingested as part of a beverage as opposed to a solid (e.g., high flavanol powder dissolved into a beverage instead of solid dark chocolate), potentially due to the faster gastric emptying associated with liquids [90]. The bioavailability and absorption of flavanols can be further improved via the simultaneous consumption of carbohydrates, as consuming ~ 4 kcal·kg^−1^ body mass alongside CF increases flavanol concentrations in the plasma by 40% [91,92]. Carbohydrates stimulate and activate sodium-glucose transport protein-1 and lactase phlorizin hydrolyase both of which are involved in flavanol absorption and metabolism [91,93]. From a practical perspective, the consumption of CF concurrently with carbohydrates post-exercise may lead to the benefits of both replenishing glycogen stores and accelerating recovery following muscle damaging exercise.

Future studies should look to investigate the muscle recovery process post EIMD alongside the supplementation of CF. A focus should be placed on whether regular supplementation of high doses of CF (> 750 mg) can affect perceived soreness, oxidative stress, and inflammation post EIMD, and whether it can influence repeat performance, fatigue, and perceived effort. Comparisons between different doses and thus establishing of an optimal dose to elicit benefits is needed before concrete recommendations can be made. It is also important that studies investigating EIMD should use protocols that evoke sufficient muscle damage (e.g., inflammation, muscle soreness). Although, such protocols may not be applicable to real world sport, they will be useful for determining the potential mechanisms by which CF might alter physiology and enhance exercise performance and recovery. Nevertheless, studies should also investigate the effect of CF supplementation on recovery following real world exercise or movements that can induce muscle damage (e.g., repeated sprint protocols) instead of solely laboratory-based protocols that may not replicate the demands or damage response that follows sporting performances. This may lead to greater practical application within sport settings. Utilising both variants of EIMD protocol approaches will aid understanding of the potential ergogenic value of supplementing CF in an athlete’s diet.

It may be pertinent to investigate prolonged flavanol supplementation on repeated bouts of exercise, with a focus on performance and recovery. Moreover, investigating the impact that CF may have on exercising muscle is required to develop greater understanding of the mechanisms in which CF exert any effects, such as their influence on endogenous antioxidant enzymes and survival signalling proteins. Indeed, future research should also look to further the knowledge of CF and their role in signalling pathways such as nuclear factor-kappa beta and nuclear erythroid 2-related factor 2, and how the regulation of these pathways may attenuate muscle damage.

## Conclusion

Few studies have examined the effects of CF on recovery following EIMD. Of the available data sub-chronic (~ 7–14 day) supplementation of CF via dark chocolate solids or in the form of a high flavanol beverage reduces exercise-induced oxidative stress and has potential for delaying fatigue during exercise allowing for prolonged performance. However, data on recovery of muscle function, and the analgesic and anti-inflammatory effects of CF is limited. Research should look to investigate these areas further to identify if CF are viable as an ergogenic aid used for recovery and potentially performance.

## Data Availability

Not applicable.

## Abbreviations

ROS: Reactive oxygen species
CF: Cocoa flavanols
EIMD: Exercise-induced muscle damage
Nrf2: Nuclear factor erythroid 2-related factor 2
MDA: Malondialdehyde
TNF-α: Tumour necrosis factor-α
IL-6: Interleukin-6
IL-10: Interleukin-10
IL-1: Interleukin-1
IL-1ra: Interleukin-1 receptor agonist
CRP: C-reactive protein
MVC: Maximal voluntary contraction
CMJ: Countermovement jump
VAS: Visual analogue scale
LEFS: Lower extremity function scale

## References

[1] Lee J, Goldfarb AH, Rescino MH, Hegde S, Patrick S, Apperson K (2002). Eccentric exercise effect on blood oxidative-stress markers and delayed onset of muscle soreness. Med Sci Sports Exerc.

[2] Powers SK, Nelson WB, Hudson MB (2011). Exercise-induced oxidative stress in humans: cause and consequences. Free Radic Biol Med.

[3] Solheim SA, Nordsborg NB, Ritz C, Berget J, Kristensen A, Mørkeberg J (2017). Use of nutritional supplements by Danish elite athletes and fitness customers. Scand J Med Sci Sport.

[4] Tangney CC, Rasmussen HE (2013). Polyphenols, inflammation, and cardiovascular disease. Curr Atheroscler Rep.

[5] Lima LJ, Almeida MH, Nout MR, Zwietering MH, Theobromacacao L (2011). “The food of the Gods”: quality determinants of commercial cocoa beans, with particular reference to the impact of fermentation. J Crit Rev Food Sci Nutr.

[6] Ortega N, Romero MP, Macia A, Reguant J, Angles N, Morello JR (2008). Obtention and characterization of phenolic extracts from different cocoa sources. J Agric Food Chem.

[7] Ioannone F, Di Mattia CD, De Gregorio M, Sergi M, Serafini M, Sacchetti GJF (2015). Flavanols, proanthocyanidins and antioxidant activity changes during cocoa (*Theobroma cacao* L.) roasting as affected by temperature and time of processing. Food Chem.

[8] Meng CC, Jalil AM, Ismail A (2009). Phenolic and theobromine contents of commercial dark, milk and white chocolates on the Malaysian market. Molecules.

[9] Andres-Lacueva C, Monagas M, Khan N, Izquierdo-Pulido M, Urpi-Sarda M, Permanyer J (2008). Flavanol and flavonol contents of cocoa powder products: influence of the manufacturing process. J Agric Food Chem.

[10] Heiss C, Finis D, Kleinbongard P, Hoffmann A, Rassaf T, Kelm M (2007). Sustained increase in flow-mediated dilation after daily intake of high-flavanol cocoa drink over 1 week. J Cardiovasc Pharmacol.

[11] Berry NM, Davison K, Coates AM, Buckley JD, Howe PR (2010). Impact of cocoa flavanol consumption on blood pressure responsiveness to exercise. Br J Nutr.

[12] Horn P, Amabile N, Angeli FS, Sansone R, Stegemann B, Kelm M (2014). Dietary flavanol intervention lowers the levels of endothelial microparticles in coronary artery disease patients. Br J Nutr.

[13] Schroeter H, Heiss C, Balzer J, Kleinbongard P, Keen CL, Hollenberg NK (2006). (–)-Epicatechin mediates beneficial effects of flavanol-rich cocoa on vascular function in humans. Proc Natl Acad Sci.

[14] Ellinger S, Reusch A, Stehle P, Helfrich HP (2012). Epicatechin ingested via cocoa products reduces blood pressure in humans: a nonlinear regression model with a Bayesian approach. Am J Clin Nutr.

[15] Heiss C, Dejam A, Kleinbongard P, Schewe T, Sies H, Kelm M (2003). Vascular effects of cocoa rich in flavan-3-ols. JAMA.

[16] Bernatova I (2018). Biological activities of (−)-epicatechin and (−)-epicatechin-containing foods: focus on cardiovascular and neuropsychological health. Biotechnol Adv.

[17] Cobley JN, Close GL, Bailey DM, Davison GW (2017). Exercise redox biochemistry: conceptual, methodological and technical recommendations. Redox Biol.

[18] Prince PD, Lanzi CR, Toblli JE, Elesgaray R, Oteiza PI, Fraga CG (2016). Dietary (–)-epicatechin mitigates oxidative stress, NO metabolism alterations, and inflammation in renal cortex from fructose-fed rats. Free Radic Biol Med.

[19] Decroix L, Soares DD, Meeusen R, Heyman E, Tonoli C (2018). Cocoa flavanol supplementation and exercise: a systematic review. Sports Med.

[20] Schramm DD, Wang JF, Holt RR, Ensunsa JL, Gonsalves JL, Lazarus SA (2001). Chocolate procyanidins decrease the leukotriene-prostacyclin ratio in humans and human aortic endothelial cells. Am J Clin Nutr.

[21] Vázquez-Agell M, Urpi-Sarda M, Sacanella E, Camino-López S, Chiva-Blanch G, Llorente-Cortés V (2013). Cocoa consumption reduces NF-κB activation in peripheral blood mononuclear cells in humans. Nutr Metab Cardiovasc Dis.

[22] Vlachojannis J, Erne P, Zimmermann B, Chrubasik-Hausmann S (2016). The impact of cocoa flavanols on cardiovascular health. Phytother Res.

[23] Hoidal JR (2001). Reactive oxygen species and cell signaling. Am J Respir Cell Mol Biol.

[24] Halliwell B (2006). Phagocyte-derived reactive species: salvation or suicide?. Trends Biochem Sci.

[25] Fuchs D, Gruber A, Uberall F, Wachter H (1994). Oxidative stress and apoptosis. Immunol Today.

[26] Powers SK, Duarte J, Kavazis AN, Talbert EE (2010). Reactive oxygen species are signalling molecules for skeletal muscle adaptation. Exp Physiol.

[27] Betteridge DJ (2000). What is oxidative stress?. Metabolism.

[28] Mattson MPJA. Hormesis defined. 2008;7(1):1–7. 10.1016/j.arr.2007.08.007 PMC2248601 18162444

[29] Radak Z, Chung HY, Goto S (2008). Systemic adaptation to oxidative challenge induced by regular exercise. Free Radic Biol Med.

[30] Cheng Y-T, Wu C-H, Ho C-Y, Yen G-C (2013). Catechin protects against ketoprofen-induced oxidative damage of the gastric mucosa by up-regulating Nrf2 in vitro and in vivo. J Nutr Biochem.

[31] Paine A, Eiz-Vesper B, Blasczyk R, Immenschuh S (2010). Signaling to heme oxygenase-1 and its anti-inflammatory therapeutic potential. Biochem Pharmacol.

[32] Cordero-Herrera I, Martín MA, Goya L, Ramos S (2015). Cocoa flavonoids protect hepatic cells against high-glucose-induced oxidative stress: relevance of MAPKs. Mol Nutr Food Res.

[33] Martins TF, Palomino OM, Álvarez-Cilleros D, Martín MA, Ramos S, Goya L. Cocoa flavanols protect human endothelial cells from oxidative stress. Plant Foods Hum Nutr. 2020:1–8. 10.1007/s11130-020-00807-1 32185628

[34] Souglis A, Bogdanis GC, Chryssanthopoulos C, Apostolidis N, Geladas NDJTJOS, Research C. Time course of oxidative stress, inflammation, and muscle damage markers for 5 days after a soccer match: effects of sex and playing position. 2018;32(7):2045–54. 10.1519/JSC.0000000000002436 29309386

[35] Fisher-Wellman K, Bloomer RJ (2009). Acute exercise and oxidative stress: a 30 year history. Dyn Med.

[36] Steinbacher P, Eckl PJB (2015). Impact of oxidative stress on exercising skeletal muscle. Biomolecules.

[37] Halliwell B (2007). Biochemistry of oxidative stress.

[38] Reid MB (2001). Invited review: redox modulation of skeletal muscle contraction: what we know and what we don't. J Appl Physiol.

[39] Siems W, Capuozzo E, Lucano A, Salerno C, Crifo C (2003). High sensitivity of plasma membrane ion transport ATPases from human neutrophils towards 4-hydroxy-2, 3-trans-nonenal. Life Sci.

[40] Reid MB (2008). Free radicals and muscle fatigue: of ROS, canaries, and the IOC. Free Radic Biol Med.

[41] Fraga CG, Actis-Goretta L, Ottaviani JI, Carrasquedo F, Lotito SB, Lazarus S (2005). Regular consumption of a flavanol-rich chocolate can improve oxidant stress in young soccer players. Clin Dev Immunol.

[42] Allgrove J, Farrell E, Gleeson M, Williamson G, Cooper K (2011). Regular dark chocolate consumption’s reduction of oxidative stress and increase of free-fatty-acid mobilization in response to prolonged cycling. Int J Sport Nutr Exerc Metab.

[43] Decroix L, Tonoli C, Soares DD, Descat A, Drittij-Reijnders MJ, Weseler AR (2017). Acute cocoa Flavanols intake has minimal effects on exercise-induced oxidative stress and nitric oxide production in healthy cyclists: a randomized controlled trial. J Int Soc Sport Nutr.

[44] Wiswedel I, Hirsch D, Kropf S, Gruening M, Pfister E, Schewe T, et al. Flavanol-rich cocoa drink lowers plasma F2-isoprostane concentrations in humans. 2004;37(3):411–21. 10.1016/j.freeradbiomed.2004.05.013 15223075

[45] Decroix L, Tonoli C, Lespagnol E, Balestra C, Descat A, Drittij-Reijnders MJ (2018). One-week cocoa flavanol intake increases prefrontal cortex oxygenation at rest and during moderate-intensity exercise in normoxia and hypoxia. J Appl Physiol.

[46] Abdulkhaleq LA, Assi MA, Noor MHM, Abdullah R, Saad MZ, Taufiq-Yap YH (2017). Therapeutic uses of epicatechin in diabetes and cancer. Vet World.

[47] Davison G, Callister R, Williamson G, Cooper KA, Gleeson M (2012). The effect of acute pre-exercise dark chocolate consumption on plasma antioxidant status, oxidative stress and immunoendocrine responses to prolonged exercise. Eur J Nutr.

[48] Morgan P, Wollman P, Jackman S, Bowtell JJS (2018). Flavanol-rich cacao mucilage juice enhances recovery of power but not strength from intensive exercise in healthy. Young Men.

[49] de Carvalho FG, Fisher MG, Thornley TT, Roemer K, Pritchett R, Freitas EC (2019). Cocoa flavanol effects on markers of oxidative stress and recovery after muscle damage protocol in elite rugby players. Nutrition.

[50] Taub PR, Ramirez-Sanchez I, Patel M, Higginbotham E, Moreno-Ulloa A, Roman-Pintos LM (2016). Beneficial effects of dark chocolate on exercise capacity in sedentary subjects: underlying mechanisms. A double blind, randomized, placebo controlled trial. Food Funct.

[51] Quindry JC, Stone WL, King J, Broeder CE (2003). The effects of acute exercise on neutrophils and plasma oxidative stress. Med Sci Sports Exerc.

[52] Glantzounis GK, Tsimoyiannis EC, Kappas AM, Galaris DA (2005). Uric acid and oxidative stress. Curr Pharm Des.

[53] Ghezzi P (2020). Environmental risk factors and their footprints in vivo—a proposal for the classification of oxidative stress biomarkers. Redox Biol.

[54] El Ridi R, Tallima H (2017). Physiological functions and pathogenic potential of uric acid: a review. J Adv Res.

[55] Mohos V, Pánovics A, Fliszár-Nyúl E, Schilli G, Hetényi C, Mladěnka P (2019). Inhibitory effects of quercetin and its human and microbial metabolites on xanthine oxidase enzyme. Int J Mol Sci.

[56] Radak Z, Taylor AW, Ohno H, Goto S (2001). Adaptation to exercise-induced oxidative stress: from muscle to brain. Exerc Immunol Rev.

[57] Peternelj TT, Coombes JS (2011). Antioxidant supplementation during exercise training: beneficial or detrimental?. Sports Med.

[58] Martinez-Negrin G, Acton JP, Cocksedge SP, Bailey SJ, Clifford T. The effect of dietary (poly) phenols on exercise-induced physiological adaptations: a systematic review and meta-analysis of human intervention trials. Crit Rev Food Sci Nutr. 2020:1–16. 10.1080/10408398.2020.1860898 33356471

[59] Peake JM, Neubauer O, Della Gatta PA, Nosaka K (2017). Muscle damage and inflammation during recovery from exercise. J Appl Physiol.

[60] Kanda K, Sugama K, Hayashida H, Sakuma J, Kawakami Y, Miura S (2013). Eccentric exercise-induced delayed-onset muscle soreness and changes in markers of muscle damage and inflammation. Exerc Immunol Rev.

[61] Kasapis C, Thompson PD (2005). The effects of physical activity on serum C-reactive protein and inflammatory markers: a systematic review. J Am Coll Cardiol.

[62] Peake J, Nosaka KK, Suzuki K. Characterization of inflammatory responses to eccentric exercise in humans. 2005. 16385845

[63] Paulsen G, Crameri R, Benestad HB, Fjeld JG, Mørkrid L, Hallén J (2010). Time course of leukocyte accumulation in human muscle after eccentric exercise. Med Sci Sports Exerc.

[64] Kim J-E, Son JE, Jung SK, Kang NJ, Lee CY, Lee KW (2010). Cocoa polyphenols suppress TNF-α-induced vascular endothelial growth factor expression by inhibiting phosphoinositide 3-kinase (PI3K) and mitogen-activated protein kinase kinase-1 (MEK1) activities in mouse epidermal cells. Br J Nutr.

[65] Rodríguez-Ramiro I, Ramos S, López-Oliva E, Agis-Torres A, Bravo L, Goya L (2013). Cocoa polyphenols prevent inflammation in the colon of azoxymethane-treated rats and in TNF-α-stimulated Caco-2 cells. Br J Nutr.

[66] Sarriá B, Martínez-López S, Sierra-Cinos JL, García-Diz L, Mateos R, Bravo L (2014). Regular consumption of a cocoa product improves the cardiometabolic profile in healthy and moderately hypercholesterolaemic adults. Br J Nutr.

[67] Esser D, Mars M, Oosterink E, Stalmach A, Müller M, Afinan LA (2014). Dark chocolate consumption improves leukocyte adhesion factors and vascular function in overweight men. FASEB J.

[68] Goya L, Martín MÁ, Sarriá B, Ramos S, Mateos R, Bravo L (2016). Effect of cocoa and its flavonoids on biomarkers of inflammation: studies of cell culture, animals and humans. Nutrients.

[69] Ellinger S, Stehle PJN (2016). Impact of cocoa consumption on inflammation processes—a critical review of randomized controlled trials. Nutrients.

[70] Murphy KJ, Chronopoulos AK, Singh I, Francis MA, Moriarty H, Pike MJ (2003). Dietary flavanols and procyanidin oligomers from cocoa (Theobroma cacao) inhibit platelet function. Am J Clin Nutr.

[71] Selmi C, Mao TK, Keen CL, Schmitz HH, Eric Gershwin M. The anti-inflammatory properties of cocoa flavanols. J Cardiovasc Pharmacol. 2006;47 Suppl 2:S163–71; discussion S72–6. 10.1097/00005344-200606001-00010 16794453

[72] Malm C, Yu JG (2012). Exercise-induced muscle damage and inflammation: re-evaluation by proteomics. Histochem Cell Biol.

[73] Deng B, Wehling-Henricks M, Villalta SA, Wang Y, Tidball JG (2012). IL-10 triggers changes in macrophage phenotype that promote muscle growth and regeneration. J Immunol.

[74] Metsios GS, Moe RH, Kitas GD. Exercise and inflammation. Best Pract Res Clin Rheumatol. 2020:101504. 10.1016/j.berh.2020.101504 32249021

[75] Kang C, Ji LL (2012). Role of PGC-1α signaling in skeletal muscle health and disease. Ann N Y Acad Sci.

[76] Clifford T, Jeffries O, Stevenson EJ, Davies KAB (2020). The effects of vitamin C and E on exercise-induced physiological adaptations: a systematic review and Meta-analysis of randomized controlled trials. Crit Rev Food Sci Nutr.

[77] Myburgh KH (2014). Polyphenol supplementation: benefits for exercise performance or oxidative stress?. Sports Med.

[78] Beyer KS, Stout JR, Fukuda DH, Jajtner AR, Townsend JR, Church DD (2017). Impact of polyphenol supplementation on acute and chronic response to resistance training. J Strength Cond Res.

[79] Peschek K, Pritchett R, Bergman E, Pritchett K (2013). The effects of acute post exercise consumption of two cocoa-based beverages with varying flavanol content on indices of muscle recovery following downhill treadmill running. Nutrients.

[80] Corr LD, Field A, Pufal D, Killey J, Clifford T, Harper LD (2020). Acute consumption of varied doses of cocoa flavanols does not influence exercise-induced muscle damage. Int J Sport Nutr Exerc Metab.

[81] Bowtell J, Kelly V (2019). Fruit-derived polyphenol supplementation for athlete recovery and performance. Sports Med.

[82] Paulsen G, Ramer Mikkelsen U, Raastad T, Peake JMJE. Leucocytes, cytokines and satellite cells: what role do they play in muscle damage and regeneration following eccentric exercise? 2012;18. 22876722

[83] Hjermstad MJ, Fayers PM, Haugen DF, Caraceni A, Hanks GW, Loge JH (2011). Studies comparing numerical rating scales, verbal rating scales, and visual analogue scales for assessment of pain intensity in adults: a systematic literature review. J Pain Symptom Manag.

[84] Yeung TS, Wessel J, Stratford P, Macdermid J (2009). Reliability, validity, and responsiveness of the lower extremity functional scale for inpatients of an orthopaedic rehabilitation ward. J Orthop Sports Phys Ther.

[85] Patel RK, Brouner J, Spendiff O (2015). Dark chocolate supplementation reduces the oxygen cost of moderate intensity cycling. J Int Soc Sports Nutr.

[86] Patel RK, Brouner J, Allgrove JE, Spendiff O (2020). The influence of different concentrations of flavanol chocolate bars under acute supplement conditions on exercise and performance. Eur J Appl Physiol.

[87] Julian R, Page RM, Harper LD. The effect of fixture congestion on performance during professional male soccer match-play: a systematic critical review with meta-analysis. Sports Med. 2020:1–19. 10.1007/s40279-020-01359-9 PMC7846542 33068272

[88] Stellingwerff T, Godin J-P, Chou CJ, Grathwohl D, Ross AB, Cooper KA (2013). The effect of acute dark chocolate consumption on carbohydrate metabolism and performance during rest and exercise. Appl Physiol Nutr Metab.

[89] Sadler D, Draijer R, Stewart CE, Jones H, Marwood S, Thijssen DH. Cocoa-flavanols enhance moderate-intensity pulmonary V̇O2 kinetics but not exercise tolerance in sedentary middle-aged adults: a randomised controlled trial. 2020. 10.1007/s00421-021-04682-9 PMC8260510 33970327

[90] Cifuentes-Gomez T, Rodriguez-Mateos A, Gonzalez-Salvador I, Alanon ME, Spencer JP (2015). Factors affecting the absorption, metabolism, and excretion of cocoa flavanols in humans. J Agric Food Chem.

[91] Schramm DD, Karim M, Schrader HR, Holt RR, Kirkpatrick NJ, Polagruto JA (2003). Food effects on the absorption and pharmacokinetics of cocoa flavanols. Life Sci.

[92] Badrie N, Bekele F, Sikora E, Sikora M (2015). Cocoa agronomy, quality, nutritional, and health aspects. Crit Rev Food Sci Nutr.

[93] Bohn T (2014). Dietary factors affecting polyphenol bioavailability. Nutr Rev.

